# Idiotic and Feeble-minded Children

**Published:** 1875-02-01

**Authors:** 


### IDIOTIC AND FEEBLE-MINDED CHILDREN.

WE have received a copy of the Memorial prepared by a committee of the Illinois State Medical Society, asking the legislature of the state to make appropriations for erecting a suitable building for the reception, training, and care of the idiotic and feeble-minded children of the state. The number of such children in the state is estimated at not less than 3000; while the old building at Jacksonville, which has been occupied for a school of this kind since 1865, accommodates only 104, and yet has on its records over 680 applicants for admission. The great value of schools for this unfortunate class has been fully demonstrated by the institutions established in Massachusetts, New York, Connecticut, Pennsylvania, Ohio, Kentucky, and Georgia.

The committee of the state society has done its work well, and no more meritorious subject will be brought to the attention of the legislature during its present session. The following is from the report of the State Board of Commissioners on Public Charities.

The Institution for Feeble-minded Children renews this winter the application made two years ago, for an appropriation of $200,000, of which $25,000 is for the purchase of a site, and $175,000 for the erection of a building designed to accommodate not less than 250 pupils.

Of this enterprise the board say: "There is no enterprise of a charitable nature in the state of Illinois which commends itself more to our sympathies than this. It seems to have been regarded by the legislature hitherto, like Bethlehem Ephrata of old, a 'little one among the thousands of Judah,' and it has been compelled to wait for its establishment upon a permanent basis until the very last. It was organized in 1865, as an experiment, under the control of the directors of the Institution for the Education of the Deaf and Dumb. In 1871, the people of the state, by their representatives, granted it an independent existence and a charter. But it still occupies leased property in the city of Jacksonville, for which it pays an annual rental of $1,000. The group of cheap wooden buildings which has sprung up around the old Duncan mansion, in which the superintendent and officers reside, is a perfect tinderbox, exposed every hour to the danger of taking fire from the stoves by which the premises are heated. Should such an accident occur, the entire structure would burn to the ground, under favorable circumstances, in fifteen minutes; and in all human probability some of the unfortunate inmates would perish miserably in the flames. Even if this were not so, the purely temporary and very inconvenient character of the accommodations, which are inadequate for the reception of more than one hundred pupils, greatly increases the aggregate as well as the *per capita* cost of the institution. It has been often and truly said that the enterprise should either be provided with better quarters or abandoned altogether. A careful observation of its practical working, during the past six years, has satisfied us of its utility and value. We have never had a doubt that the general assembly would, at some day, make a building appropriation for the benefit of the idiot school. We hope that it will do so this winter. The application for $175,000 is based upon an actual plan by an architect of ability, and upon actual estimates of cost of construction, in accordance with said plan. The sum of $25,000 for the purchase of land will perhaps not appear excessive when it is considered that the institution needs a farm of large area, both for the pasturage of cows (these children consume a great quantity of milk) and to furnish a means of physical development and practical education of the boys by farm labor, and that this farm must be adjacent to or not far from some large town."

